# Places to purchase food in urban and rural areas of Brazil

**DOI:** 10.1590/1980-549720240047

**Published:** 2024-10-11

**Authors:** Thais Meirelles de Vasconcelos, Kesya Santos Felix Pereira, Jamile Carvalho Tahim, Rosely Sichieri, Ilana Nogueira Bezerra

**Affiliations:** I Universidade Estadual do Ceará, Postgraduate Program in Collective Health – Fortaleza (CE), Brazil.; II Universidade Estadual do Ceará, Nutrition Course, Health Sciences Center – Fortaleza (CE), Brazil.; III Universidade do Estado do Rio de Janeiro, Institute of Social Medicine – Rio de Janeiro (RJ), Brazil.; IV Universidade Estadual do Ceará, Postgraduate Program in Nutrition and Health – Fortaleza (CE), Brazil.

**Keywords:** Eating habits, Consumer behavior, Food social space, Diet surveys

## Abstract

**Objective:**

To describe the locations of food and beverage acquisition in Brazil, according to the level of food processing and household location (urban/rural).

**Methods:**

Data from 49,489 households from the Household Budget Survey 2017-2018 were used. Information regarding food and beverages was collected through a collective acquisition booklet over 7 consecutive days. Locations were classified into 10 groups on the basis of similarities in sales structure and mode of food offering, and food and beverages were categorized according to the NOVA classification. The frequency of households acquiring food in each location was estimated, as well as the acquisition frequency according to processing level, considering significance through non-overlapping 95% confidence intervals.

**Results:**

Half of the households (51.9%) acquired food in supermarkets, contributing to both the acquisition of fresh and minimally processed foods (92.2% in urban; 90.2% in rural) and ultra-processed foods (78.6% in urban; 74.1% in rural). For the urban area, the Supermarket (55.0%), Bakery (46.5%) and Small markets (43.1%) are among the places with the highest frequency of food acquisition, while for rural areas, it is found that Small Markets (53%), Supermarkets (32.3%) and Home production (31.0%) presented the highest frequencies.

**Conclusion:**

The acquisition of food and beverages for household consumption in Brazil differs according to household location (urban/rural), indicating the importance of the community food environment in the consumption patterns of ultra-processed foods.

## INTRODUCTION

The food environment comprises the physical space, economic, political and collective sociocultural factors, as well as opportunities and conditions that drive food and beverage choices, where individuals’ nutritional status is directly impacted^1^. It is the connecting field where people interact with the broader food system to acquire and consume food^2^. Factors such as availability and accessibility have been consistently identified as determinants of individuals’ choice of location and purchasing behaviors^3^.

Industrialization, urbanization and globalization have had a major impact on food production and distribution methods, leading to changes in the way people access them^4^. More traditional and smaller markets, such as fruit and vegetable markets, street markets and small grocery stores, which used to be the most important places for purchasing food, have been losing ground in this sector, while at the same time, supermarkets and hypermarkets have been gaining a greater share^5^.

Studies on food purchasing sites in Brazil have shown a preference for purchasing food in supermarkets, and some studies indicate that fresh food markets, such as fairs, have lost importance and been replaced by supermarkets^6,7^. Data from the Household Budget Survey (POF) showed that supermarkets/hypermarkets were responsible for the majority of food purchases in Brazil, contributing significantly to the purchase of ultra-processed products^5,8^. The mapping of food deserts carried out by the Interministerial Chamber for Food and Nutrition Security (CAISAN) in 2018 is another important source of information on the food environment in the country; however, it is restricted to the formal labor market and does not assess the purchases of each household, failing to include food produced at home purchased by vendors and places whose main purpose is not to sell food^9^. In addition, changes in the consumption patterns of households located in rural areas of the country have been pointing to a new context of rural life, with eating habits more similar to those in urban areas^10,11^.

Given the above, it can be seen that the food environment is an important factor in the study of food consumption, and is a potential target for the promotion of practices that are capable of influencing healthy eating^3,12^. Thus, the objective of the present study was to describe the places of food and beverage purchase in Brazil, according to the level of processing and the area of residence (urban and rural), based on data from the POF carried out in 2017–2018.

## METHODS

This is a cross-sectional study, and the data used in this study come from the 2017–2018 POF, carried out by the Brazilian Institute of Geography and Statistics (IBGE), which aims to measure the composition of household budgets and the living conditions of the Brazilian population^13^. The POF sampling plan is carried out by cluster in two stages. The Primary Sampling Units (UPA) correspond to census sectors or aggregates of sectors randomly selected from a master sample. The secondary units correspond to households randomly selected within the sectors. The sectors underwent geographic and statistical stratification based on the income of the head of the household from the 2010 Demographic Census^13^, allowing estimates to be generated for different geographic strata (urban and rural areas, five Brazilian regions, metropolitan areas of the capitals) and socioeconomic levels^13^. For the 2017–2018 POF, 5,504 UPAs and 75,635 households were selected, and interviews were conducted in 57,920 households^11^. For this study, all households that responded to the collective acquisition booklet with information on the acquisition of food and beverages were included in the analyses (n=49,489). Details of the sampling procedure can be found at: https://www.ibge.gov.br/estatisticas/sociais/saude/24786-pesquisa-de-orcamentos-familiares-2.html.

Information on food and beverages purchased by families was recorded daily and for seven consecutive days, with a detailed description of each product purchased, quantity, unit of measurement with its equivalent in weight or volume, the amount of the expense in reais, the place of purchase and the method of purchase of the product^13^. This study considered the concept of community food environment and, as a dimension, the types of establishments where food and beverages were purchased in Brazil^14^. The records relating to the places where food was purchased were classified and grouped into the ten categories below, according to the similarities in the sales structure and the way food was offered, seeking to follow patterns proposed in other studies^11,15^.

1. Supermarket — supermarket, hypermarket, warehouse;
2. Bakery — bakery, bread warehouse, confectionary, delicatessen;
3. Small markets — grocery store, bodega, shop, small warehouse, minibox shop;
4. Fruit store/fair/street market — green grocer, vegetable market, street market, organic market, fruit shop;
5. Butcher’s/fish market — butcher, meatpacking plant, slaughterhouse, fish market, poultry farm;
6. Street food/street vendors —formal or informal street vendors, trailer or kiosk, beach and street stalls;
7. Restaurant/diner/bar and grill/cafeteria — canteen, pastry shop, pizzeria, snack shop, ice cream shop, bar, self-service;
8. Home production — farm, garden, community garden, rural producer, own business, yard stand;
9. Food distribution centers/wholesalers — wholesale warehouse, supply centers, food distributor, state market;
10. Others — places whose main purpose is not selling food such as churches, bazaars, exhibition fairs, veterinary clinics, clothes stores, etc.

All food and beverages purchased, considered in this study, were categorized according to the NOVA classification, which encompasses four groups, according to the level of processing: fresh or minimally processed foods, culinary ingredients, processed and ultra-processed foods^16^.

Initially, the characteristics of the households participating in the study (region, location area [urban and rural] and per capita family income) were described through absolute and relative frequencies. Per capita family income was stratified based on the minimum wage in effect in January 2018 (R$ 954.00; US$ 297.00): less than one minimum wage, one to two, two to three and more than four minimum wages.

The frequency of households that purchased food in each of the locations was estimated according to the places of purchase and the level of processing of the food purchased (fresh, ingredients, processed or ultra-processed). For both estimates, the purchase of at least one item at the place of purchase or one item from the food group was considered. To estimate the frequency of acquisition of food groups according to the place of acquisition, the number of households that acquired at least one food item from a food group at a given place of acquisition was divided by the number of households that acquired at least one item at that place.

The data were presented as relative frequencies, and the significance of differences was considered by means of non-overlapping 95% confidence intervals (95%CI). All estimates were stratified according to the location of the household (urban/rural) and per capita family income ranges, calculated by the SAS software, and took into account the complexity of the design and the sample weight of the survey.

## RESULTS

Of the 49,489 households assessed, 82.3% were located in urban areas of the country; in 51.4%, the head of the family had completed elementary school; and 69.3% had a per capita family income of up to two minimum wages (Table 1).

**Table 1:** Sociodemographic characteristics of households. Brazil, 2017–2018.

Variables	2017–2018
	% (95%CI)
Location area	
Urban	82.3 (85.7–86.8)
Rural	13.7 (13.2–14.2)
Region	
North	7.1 (6.9–7.4)
Northeast	27.7 (27.1–28.3)
Southeast	42.2 (41.5–43.0)
South	15.4 (14.9–15.9)
Central-West	7.5 (7.1–7.9)
Education*	
Elementary school	51.4 (50.6–52.3)
Secondary school	29.6 (29.0–30.3)
Higher education	18.9 (18.1–19.8)
Income (minimum wage^)†^	
≤1	34.2 (33.5–35.0)
1–2	35.1 (34.4–35.9)
2–4	13.9 (13.4–14.4)
>4	16.7 (15.8–17.5)

*Education of head of the family;

^†^Per capita household income. CI: confidence interval.

The places that contributed most to the purchase of food/beverages were supermarkets (51.9%), followed by small markets (44.5%) and bakeries (43.8%). Food distribution centers/wholesalers had the lowest share (5.5%). Significant differences between urban and rural areas were found. For urban areas, supermarkets (55.0%), bakeries (46.5%) and small markets (43.1%) were among the places with the highest frequency of food purchase, while for rural areas, it was found that small markets (53%), supermarkets (32.3%) and home production (31.0%) had the highest frequencies (Table 2).

**Table 2:** Frequency (%) and confidence interval of food/beverage purchase places according to area (urban/rural). Brazil, 2017–2018.

Acquisition place*	Brazil (%) (95%CI)	Urban (%) (95%CI)	Rural (%) (95%CI)
Supermarket	51.9 (51.0–52.8)	55.0 (54.0–56.0)	32.3 (30.5–34.2)
Bakery	43.8 (43.0–44.6)	46.5 (45.6–47.4)	26.7 (25.0–28.3)
Small market	44.5 (43.6–45.4)	43.1 (42.1–44.1)	53.0 (50.8–55.1)
Fruit stand/fair/street market	23.0 (22.3–23.6)	23.2 (22.5–24.0)	21.3 (19.4–23.1)
Butcher’s/fish market	16.3 (15.8–16.9)	16.0 (15.4–16.6)	18.5 (17.2–19.4)
Street food/street vendor	9.5 (6.1–9.9)	8.2 (7.8–8.7)	17.5 (15.8–19.3)
Restaurant/snack bar/bar and grill/cafeteria	7.2 (6.8–7.6)	7.8 (7.3–8.2)	3.8 (3.2–4.3)
Home production	7.0 (6.6–7.4)	3.2 (2.9–3.4)	31.0 (28.7–33.3)
Food distribution center/wholesaler	5.5 (5.1–5.8)	5.9 (5.5–6.4)	2.4 (1.9–3.0)
Others	11.2 (10.8–11.7)	10.0 (9.5–10.4)	19.2 (17.8–20.6)

*% of households that purchased at least one food/beverage item from that place. CI: confidence interval.

**Figure 1:**
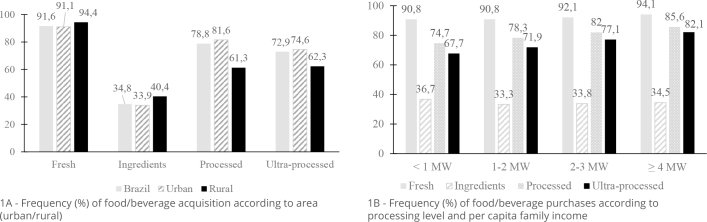
Frequency (%) of food/beverage acquisition according to the level of processing of the food acquired. Brazil, 2017–2018.

With regard to the frequency of food/beverage acquisition according to the level of processing, 91.6% of the households analyzed bought fresh foods, followed by 78.8% processed foods and 72.9% ultra-processed foods. Ingredients accounted for only 34.8%. High frequency percentages for processed and ultra-processed foods were observed among households in urban areas of the country, and the purchase of ultra-processed foods ranked second among rural households (Figure 1A). Regarding income, there was no significant difference for the acquisition of fresh foods and ingredients in the first three income levels, but the acquisition of processed and ultra-processed foods increased with income (Figure 1B).

The places where food was purchased also differed according to income level, with purchases in supermarkets being 29.6 percentage points higher among those earning more than four minimum wages compared to those earning less than one. On the other hand, the percentage of food purchased from bakeries, small markets, butcher’s/fish market, street food/street vendors and home-produced foods decreased with income (Table 3).

**Table 3:** Frequency (%) and confidence interval of food/beverage purchase places according to household income per capita. Brazil, 2017–2018.

Acquisition place*	≤1 MW % (95%CI)	1–2 MW % (95%CI)	2–4 MW % (95%CI)	>4 MW % (95%CI)
Supermarket	39.7 (38.5–40.8)	52.3 (51.0–53.6)	60.0 (58.0–62.0)	69.3 (67.5–71.3)
Bakery	47.7 (46.6–48.8)	44.5 (43.3–45.6)	39.4 (37.4–41.5)	37.9 (36.0–39.8)
Small markets	54.9 (53.7–56.1)	43.0 (41.8–44.2)	38.5 (36.6–40.4)	31.2 (29.2–33.2)
Fruit stand/fair/street market	23.4 (22.4–24.5)	22.5 (21.6–23.5)	22.4 (20.7–24.2)	23.2 (21.6–24.8)
Butcher’s/fish market	19.6 (18.7–20.4)	16.0 (15.2–16.8)	14.4 (13.1–15.7)	12.0 (10.8–13.2)
Street food/street vendor	12.8 (12.0–13.6)	9.2 (8.6–9.8)	7.0 (6.2–7.9)	5.5 (4.8–6.3)
Restaurant/snack bar/bar and grill/cafeteria	5.3 (4.8–5.8)	7.1 (6.5–7.7)	8.7 (7.5–9.9)	10.2 (9.2–11.3)
Home production	9.2 (8.5–9.8)	7.3 (6.7–7.8)	5.5 (4.8–6.1)	3.1 (2.7–3.6)
Food distribution center/wholesaler	4.1 (3.7–4.6)	5.5 (4.9–6.1)	6.6 (5.3–7.9)	7.1 (6.2–8.0)
Others	12.6 (11.9–13.3)	10.7 (10.0–11.3)	9.9 (8.9–10.9)	10.6 (9.5–11.7)

*% of households that purchased at least one food/beverage item from that place. MW; minimum wage; CI: confidence interval.

For urban areas, most fresh foods were purchased from butcher’s/fish market (97.5%), fruit shop/fairs/street market (97.1%), home-produced foods (95.3%) and supermarkets (92.2%). Processed foods were mostly purchased from bakeries. Regarding the ultra-processed group, the place with the highest frequency of purchase was the supermarket (78.6%) (Table 4).

**Table 4:** Frequency (%) and confidence interval of acquisition of food groups according to acquisition places in the urban area. Brazil, 2017–2018.

Acquisition place*	Fresh (95%CI)	Ingredients (95%CI)	Processed (95%CI)	Ultra-processed (95%CI)
Supermarket	92.2 (91.6–92.7)	37.5 (36.4–38.5)	61.0 (59.9–62.1)	78.6 (77.7–79.5)
Bakery	21.0 (20.0–22.0)	1.0 (0.8–1.2)	95.5 (95.1–96.0)	28.3 (27.3–29.3)
Small markets	84.4 (83.5–85.2)	26.7 (25.7–27.7)	49.1 (47.9–50.3)	67.1 (66.1–68.1)
Fruit stand/fair/street market	97.1 (96.6–97.6)	2.5 (2.1–3.0)	10.6 (9.4–11.9)	12.3 (11.2–13.5)
Butcher’s/fish market	97.5 (97.0–98.1)	0.4 (0.2–0.6)	3.3 (2.7–3.9)	14.6 (13.2–16.1)
Street food/street vendor	82.1 (80.0–84.3)	0.5 (0.3–0.8)	18.2 (16.0–20.3)	11.6 (9.9–13.4)
Restaurant/snack bar/bar and grill/cafeteria	43.3 (40.6–46.0)	0.3 (0.0–0.6)	18.6 (16.5–20.7)	60.5 (57.6–63.4)
Home production	95.3 (93.6–96.9)	5.4 (3.5–7.2)	10.2 (8.2–12.3)	8.8 (6.6 –10.9)
Food distribution center/wholesaler	82.8 (80.3–85.3)	25.1 (22.3–27.8)	40.3 (37.0–43.7)	51.8 (48.5–55.0)
Others	69.6 (67.4–71.8)	6.0 (5.0–7.0)	23.7 (21.8–25.6)	34.0 (31.9–36.1)

*% of households that purchased at least one food/drink item from that location local. CI: confidence interval.

In rural areas, the main place for purchasing fresh foods was home production (98.8%), followed by butcher’s/fish market (97.9%) and fruit shop/fair/street market (96.5%), but the acquisition of culinary ingredients, processed and ultra-processed foods also took place largely in supermarkets and small markets (Table 5).

**Table 5:** Frequency (%) and confidence interval of acquisition of food groups according to acquisition places in rural areas. Brazil, 2017–2018.

Acquisition place*	Fresh (95%CI)	Ingredients (95%CI)	Processed (95%CI)	Ultra-processed (95%CI)
Supermarket	90.2 (89.0–91.5)	49.0 (46.6–51.5)	44.6 (42.1–47.2)	74.1 (71.9–76.2)
Bakery	11.7 (9.9–13.5)	1.1 (0.4–1.7)	95.3 (94.3–96.3)	18.8 (16.7–20.9)
Small markets	85.5 (84.0–87.0)	41.3 (39.3–43.3)	38.4 (36.6–40.5)	64.0 (62.1–65.8)
Fruit stand/fair/street market	96.5 (95.2–97.8)	7.5 (5.5–9.4)	9.6 (7.2–12.0)	14.9 (11.9–17.9)
Butcher’s/fish market	97.9 (97.1–98.6)	0.5 (0.03–1.0)	1.6 (0.7–2.4)	6.7 (5.2–8.3)
Street food/street vendor	69.9 (65.4–74.5)	1.6 (0.8–2.3)	43.2 (37.4–49.1)	10.4 (8.3–12.5)
Restaurant/snack bar/bar and grill/cafeteria	37.5 (30.6–44.5)	3.7 (1.2–6.1)	29.7 (23.7–35.6)	54.5 (47.8–61.1)
Home production	98.8 (98.3–99.3)	1.7 (1.2–2.3)	6.1 (4.8–7.4)	3.6 (2.7–4.5)
Food distribution center/wholesaler	85.8 (79.8–91.9)	39.8 (31.7–48.0)	35.9 (26.6–45.2)	43.8 (32.6–55.0)
Others	86.6 (84.4–88.9)	6.1 (4.7–7.5)	18.0 (15.1–20.8)	16.7 (14.4–18.9)

*% of households that purchased at least one food/beverage item from that place. CI: confidence interval.

## DISCUSSION

This study determined the places of food purchase according to the level of processing and the area where the household is located (urban/rural), based on data from the POF carried out in 2017–2018. The places that had the greatest participation in the acquisition of food and beverages were supermarkets, small markets and bakeries, with differences between urban and rural areas. The supermarket remained the place that contributed the most for obtaining food and beverages in urban areas, while for rural areas, it was the small markets. Home production was in third place in relation to the places of acquisition in rural areas and was ten times higher than the frequency in urban areas.

Regarding the frequency of acquisition of food/beverage groups, it is observed that the purchase of culinary ingredients and processed and ultra-processed foods took place largely in supermarkets and small markets, both in urban and rural areas. However, it is noteworthy that for rural areas, the main place for obtaining fresh foods was home production.

The differences between urban and rural areas of the country may be a reflection of the urbanization process in the regions, which influences the food consumption of the Brazilian population. In this scenario, urban areas demonstrate repercussions on eating habits with greater adherence to processed and ultra-processed items due to lifestyle and the search for the convenience of ready-to-eat items. In parallel to this, there is a tendency for integration of consumption patterns with the advance of globalization in view of the opportunities for access to information and availability of industrialized items in rural areas^17^. The urbanization of rural areas, promoted by the growing industrialization process and expanded by agri-food production, has contributed to the change in lifestyle and, consequently, in eating patterns^18^. Analyses of the temporal trend of changes in the consumption of ultra-processed foods found an increase in the participation of these items in the diet of individuals in rural areas compared to urban areas between 1974 and 2018 (2.43; 95%CI 2.0–2.87 vs. 0.6; 95%CI 0.23–0.98, respectively)^10^.

Different determinants are related to dietary patterns, which can be physical, economic, political, cultural or social in nature, and do not correspond solely to issues of individual choice^4^. Studies show that income is associated with an important determinant of food choice and acquisition patterns, highlighting that access to and consumption of foods considered healthy are higher among individuals with higher socioeconomic status. Conversely, individuals with greater socioeconomic vulnerability have less access to healthy items, such as fruits and vegetables, and greater exposure to processed and ultra-processed items^19-21^.

Differences in income level were also observed in our study, with the frequency of supermarket purchases being almost 30% higher among individuals with higher socioeconomic status. In addition, households with higher incomes have higher frequencies of purchases of both fresh and processed and ultra-processed foods. Levy et al.^10^ found that the higher the income, the greater the share of ultra-processed foods in the total energy consumed. However, when evaluating the temporal trend of changes in the consumption of ultra-processed foods, they observed a more significant increase in the share of the diet of individuals with lower socioeconomic status.

Inequalities in the availability of and access to the food environment in which individuals live contribute to the increase in food insecurity in economically disadvantaged areas. This is because regions with lower socioeconomic status are often characterized as food swamps, places where there is a greater availability of establishments that sell processed and ultra-processed items. This scenario limits access to foods considered markers of healthy eating and increases exposure to items whose regular consumption is widely related to unfavorable health and nutrition outcomes^22^.

We observed that the supermarket was an environment that contributed significantly to the acquisition of ultra-processed foods, suggesting that these items are highly available in the consumer environment. In addition to our findings, national studies that evaluated the relationship between food consumption and the place where food was purchased observed that access to items from supermarkets was associated with the consumption of foods with low nutritional quality, such as ultra-processed foods^11,14^. Small markets also contributed significantly to food acquisition and were associated with the availability of ultra-processed items, which are often attractive because they are easier to store and do not require specific equipment for marketing^23^.

The high acquisition of ultra-processed foods may be related to both price and ease of consumption and the appeal of promotions. A study that sought to relate the prevalence of price promotions on food and beverages and their influence on consumer purchasing behavior found that price promotions were more common for unhealthy foods and beverages^24^. Living in areas far from food shopping locations is also associated with a higher consumption of ultra-processed foods, while buying food from establishments closer to home is associated with a healthier eating pattern^23^. On the other hand, living in areas where there are fairs and markets that sell good quality fruits, vegetables and legumes makes it more likely to adopt healthy eating patterns^3^.

Although most households purchased fresh foods (91.6%), we also observed a high level of acquisition of processed and ultra-processed items, 78.8 and 72.9%, respectively. In addition to availability, accessibility, convenience, price, promotions and quality, other determinants can influence food acquisition behavior and changes in consumption patterns, such as sociocultural characteristics, convenience, time and effort savings and sustainability of items in certain environments^25^. Studies conducted in Brazil reinforce our findings by identifying an increase in the consumption pattern of items that are markers of healthy eating and stability in the consumption of items considered markers of unhealthy eating, such as fast-food combinations, sweets and cookies, packaged snacks and soft drinks^26^. The adoption of an unhealthy pattern can be related not only to the dimensions of the community food environment, but also to changes in daily life and in the ways in which food prepared outside the home is acquired. Although restaurants/snack bars/bars/cafeterias had a low frequency of purchase, it was observed that food purchase in these establishments included ultra-processed foods, which may be an indication of the purchase of ready-to-eat foods prepared outside the home. The comparison of POF data from 2002–03 to 2017–18 shows an increase in the purchase of ready-to-eat meals in Brazilian households, rising from 0.2 to 0.7%, respectively^27^. These foods can be consumed both in the establishments and in the home through purchase via delivery or takeaway^8^. These purchasing practices have become part of individuals’ daily lives and are considered a determining factor in the increase in overweight and obesity levels due to their unfavorable nutritional content^8^.

More recently, the practice of purchasing food prepared outside the home has also seen an increase in the use of digital device applications. Studies on the availability of food in the digital food environment of food delivery apps appear to show similarities to the characteristics of a food swamp, due to the high availability of items such as snacks and fast-food combinations and sugary drinks, in addition to the impact of marketing and loyalty strategies to influence consumer choices^28-30^.

The recommendations of the Food Guide for the Brazilian Population advise that the basis of the diet should be composed of fresh or minimally processed foods, foods that provide a nutritionally adequate and tasty diet and recognize the relevance of cultural and sustainability aspects involving food. They encourage the purchase of food from street markets, local producers or other places with an agroecological base^16^. Strengthening the production of local farmers and valuing regional inputs are strategies that can favor healthier and more sustainable eating patterns^31^.

The Brazilian government’s efforts to overcome barriers in predominantly unhealthy consumer food environments stand out to increase the population’s access to healthier foods. One example is the change in the composition of the Brazilian basic food basket, favoring fresh and minimally processed items, taking into account the nutritional needs of the different regions of the country, focusing on family farming and excluding ultra-processed foods. The regulation of the Cozinha Solidária Program, an initiative aimed at distributing food free of charge or at affordable prices, is another strategy that can contribute to improving food and nutritional security and combating hunger in the country, providing access to better quality food^32^. On the other hand, changes in the environment require intersectoral public policies^33^. Examples include Mexico and the United Kingdom, which adopted policies to tax sugary drinks, and the first results in Mexico showed that this change led to a 6% reduction in the purchase of these drinks^34,35^.

An important piece of information to be analyzed in our findings is the category of places of purchase called “Others”, that is, places that did not have the main purpose of selling food. Thus, it is believed that such places sell products that are easy to preserve, such as ultra-processed foods. However, it was observed that households that purchased food in these places bought more fresh foods, representing a positive finding observed in the present study. Currently, many foods can be purchased via the internet, but this was not considered a specific place of purchase for this study because it represents only a means of acquiring the product, which can come from any place previously characterized, and there is no way to distinguish it, which represents a limitation of this study. Added to this limitation is the lack of detailed information about the places of purchase, for example, whether the distribution centers are public Food and Nutrition Security facilities that distribute/donate food. However, “food distribution centers” were reported and included in this analysis. Furthermore, we considered all monetary and non-monetary acquisitions, so items acquired through donations or through access to benefits were included in the analyses.

As a strong point, this article is the most up-to-date regarding the source of food acquired for consumption in Brazilian households, being able to outline an overview of food acquisition and consumption practices, guiding the development of strategies and interventions that are capable of providing healthier food environments, improving food and nutritional security and preventing diseases associated with lifestyle.

In terms of public health, it is worth highlighting the importance of the differences observed between urban and rural areas, indicating the need for strategies involving multiple components to interrupt the inversion of dietary practices in rural areas and provide better access to fresh and minimally processed foods in both rural and urban areas.

In conclusion, our findings indicate that the acquisition of food for home consumption in Brazil comes mainly from supermarkets and small markets, which despite being sources of fresh foods were also places with the highest percentage of ultra-processed foods purchased. Differences between rural and urban areas indicate the relevance of actions in the community food environment scenario, which aim at food and nutrition education processes based on the recommendations of the Food Guide for the Brazilian Population, in the multiple dimensions of healthy, sustainable food choices that value food culture in different community settings. Thus, adherence to the consumption pattern of fresh and minimally processed foods can be encouraged and the consumption of ultra-processed items discouraged.
