# Supplementary material for: Places to purchase food in urban and rural areas of Brazil
Source: Rev Bras Epidemiol. 2024 Oct 11;27:e240047. doi: 10.1590/1980-549720240047 (PMC11472665; doi:10.1590/1980-549720240047)
Supplement: Translation

# Translation: Locais de aquisição de alimentos nas áreas urbanas e rurais do Brasil

**DOI:** 10.1590/1980-549720240047.2

**Authors:** Thais Meirelles de Vasconcelos, Kesya Santos Felix Pereira, Jamile Carvalho Tahim, Rosely Sichieri, Ilana Nogueira Bezerra

**Affiliations:** I Universidade Estadual do Ceará, Programa de Pós-Graduação em Saúde Coletiva – Fortaleza (CE), Brasil.; II Universidade Estadual do Ceará, Curso de Nutrição, Centro de Ciências da Saúde – Fortaleza (CE), Brasil.; III Universidade do Estado do Rio de Janeiro, Institute of Social Medicine – Rio de Janeiro (RJ), Brasil.; IV Universidade Estadual do Ceará, Programa de Pós-Graduação em Nutrição e Saúde – Fortaleza (CE), Brasil.

**Keywords:** Hábito alimentar, Comportamento do consumidor, Ambiente alimentar, Inquéritos alimentares

## Abstract

**Objetivo:**

Descrever os locais de aquisição de alimentos e bebidas consumidos no Brasil, conforme o nível de processamento dos alimentos e a área de localização do domicílio (urbano/rural).

**Métodos:**

Utilizaram-se dados de 49.489 domicílios da Pesquisa de Orçamentos Familiares 2017–2018. As informações relativas aos alimentos e bebidas foram coletadas por meio da caderneta de aquisição coletiva durante sete dias consecutivos. Os locais de aquisição foram classificados em dez grupos de acordo com as similaridades na estrutura de venda e no modo de oferta de alimentos, e todos os alimentos e bebidas foram categorizados de acordo com a classificação NOVA. Estimou-se a frequência de domicílios que adquiriram alimentos em cada um dos locais, bem como a frequência de aquisição segundo o nível de processamento, e considerou-se a significância por meio da não sobreposição dos intervalos de confiança de 95%.

**Resultados:**

Metade dos domicílios (51,9%) adquiriu alimentos em supermercados, locais estes que contribuíram tanto para a aquisição de alimentos *in natura* (92,2% urbano; 90,2% rural) quanto ultraprocessados (78,6% urbana; 74,1% rural). Para a área urbana, supermercado (55,0%), padaria (46,5%) e pequenos mercados (43,1%) estão entre os locais com maior frequência de aquisição de alimentos, enquanto, para as áreas rurais, verifica-se que os pequenos mercados (53%), supermercado (32,3%) e produção em casa (31,0%) apresentaram as maiores frequências.

**Conclusão:**

A aquisição de alimentos e bebidas para consumo domiciliar no Brasil difere de acordo com a área de localização do domicílio (urbana/rural), indicando a importância do cenário do ambiente alimentar comunitário nos padrões de consumo de alimentos ultraprocessados.

## INTRODUÇÃO

O ambiente alimentar compreende o espaço físico, fatores econômicos, políticos e socioculturais coletivos, bem como oportunidades e condições que induzem as escolhas de alimentos e bebidas, impactando diretamente o estado nutricional dos indivíduos^1^. É o campo de conexão onde as pessoas interagem com o sistema alimentar mais amplo para adquirir e consumir alimentos^2^. Fatores como disponibilidade e acessibilidade foram consistentemente identificadas como determinantes na escolha do local e dos comportamentos de compra dos indivíduos^3^.

A industrialização, a urbanização e a globalização tiveram grande impacto nos métodos de produção e distribuição de alimentos, gerando mudanças na forma de acesso a eles^4^. Mercados mais tradicionais e menores, como hortifrutigranjeiros, feiras livres e pequenas mercearias, que costumavam ser os locais com maior importância na aquisição de alimentos, foram perdendo espaço nesse setor, enquanto, paralelamente a isso, os supermercados e hipermercados foram conquistando maior participação^5^.

Estudos sobre locais de aquisição de alimentos no Brasil têm mostrado preferência pela aquisição de alimentos em supermercados, e alguns estudos sinalizam que os mercados de alimentos frescos, como as feiras, têm perdido importância e sido substituídos por supermercados^6,7^. Dados da Pesquisa de Orçamentos Familiares (POF) mostraram que os super/hipermercados foram responsáveis pela maior parte das aquisições de alimentos no Brasil, contribuindo de forma importante para a compra de produtos ultraprocessados^5,8^. O mapeamento de desertos alimentares realizado pela Câmara Interministerial de Segurança Alimentar e Nutricional (CAISAN) em 2018 é outra importante fonte de informação sobre o ambiente alimentar no país; no entanto, restringe-se ao mercado formal de trabalho e não avalia as aquisições de cada domicílio, não captando alimentos produzidos em casa, adquiridos por vendedores e locais que não têm como principal finalidade a venda de alimentos^9^. Além disso, mudanças no padrão de consumo de domicílios situados na área rural do país vêm apontando um novo contexto da vida rural, com práticas alimentares mais similares com as da área urbana^10,11^.

Observa-se, diante do exposto, que o ambiente alimentar é fator importante no estudo do consumo alimentar, sendo um alvo potencial para a promoção de práticas que sejam capazes de influenciar a alimentação saudável^3,12^. Assim, o objetivo do presente estudo foi descrever os locais de aquisição de alimentos e bebidas no Brasil, segundo o nível de processamento e a área de localização do domicílio (urbano e rural), com base nos dados da POF realizada em 2017–2018.

## MÉTODOS

Trata-se de um estudo transversal, e os dados utilizados no presente estudo são provenientes da POF 2017–2018, realizada pelo Instituto Brasileiro de Geografia e Estatística (IBGE), que visa mensurar a composição dos orçamentos domésticos e as condições de vida da população brasileira^13^. O plano amostral da POF é realizado por conglomerado em dois estágios. As Unidades Primárias de Amostragem (UPA) correspondem aos setores censitários ou agregados de setores selecionados de forma aleatória de uma amostra mestra. As unidades secundárias correspondem aos domicílios selecionados de forma aleatória dentro dos setores. Os setores passaram por estratificação geográfica e estatística com base na renda do chefe da família do Censo Demográfico de 2010^13^, permitindo gerar estimativas para diferentes estratos geográficos (áreas urbana e rural, cinco regiões brasileiras, áreas metropolitanas das capitais) e níveis socioeconômicos^13^.

Para a POF de 2017–2018, foram selecionadas 5.504 UPA e 75.635 domicílios, tendo sido realizadas entrevistas em 57.920 domicílios^11^. Para este trabalho, todos os domicílios que responderam à caderneta de aquisição coletiva com informação sobre a aquisição de alimentos e bebidas foram incluídos nas análises (n=49.489). Detalhes do procedimento amostral podem ser obtidos em: https://www.ibge.gov.br/estatisticas/sociais/saude/24786-pesquisa-de-orcamentos-familiares-2.html.

As informações relativas aos alimentos e bebidas adquiridos pelas famílias foram registradas diariamente e durante sete dias consecutivos, com a descrição detalhada de cada produto adquirido, quantidade, unidade de medida com o seu equivalente em peso ou volume, o valor da despesa em reais, o local de compra e a forma de aquisição do produto^13^. O presente estudo considerou o conceito de ambiente alimentar comunitário e como dimensão os tipos de estabelecimentos de aquisição de alimentos e bebidas no território brasileiro^14^. Os registros referentes aos locais de aquisição de alimentos foram classificados e agrupados em dez categorias, de acordo com as similaridades na estrutura de venda e no modo de oferta de alimentos, buscando seguir padrões propostos em outros estudos^11,15^:

1. Supermercado — supermercado, hipermercado, armazém;
2. Padaria — padaria, depósito de pães, panificadora, confeitaria, delicatéssen;
3. Pequenos mercados — mercearia, bodega, venda, pequeno armazém, minibox;
4. Frutaria/feiras/mercado de rua — quitanda, feira de verduras, feira livre, feira orgânica, frutaria, verdureiro;
5. Açougue/peixaria — açougue, frigorífico, abatedouro, peixaria, mercado de peixes, aviário;
6. Comida de rua/vendedores de rua — vendedores de rua formais ou informais, *trailer* ou quiosque, barraca de praia e de rua;
7. Restaurante/lanchonete/bar/cafeteria — cantina, pastelaria, pizzaria, loja de salgadinhos, sorveteria, bar, *self-service*;
8. Produção em casa — fazenda, jardim, jardim comunitário, produtor rural, negócio próprio, quintal;
9. Centros de distribuição de alimentos/atacadistas — armazém atacadista, centros de abastecimento, distribuidora de alimentos, mercado estadual;
10. Outros — locais que não possuem como finalidade principal a comercialização de alimentos, como igreja, bazar, feiras de exibição, hospitais, clínicas veterinárias, lojas de roupas etc.

Todos os alimentos e bebidas adquiridos, considerados neste estudo, foram categorizados de acordo com a classificação NOVA, que abarca quatro grupos, conforme o nível de processamento: alimentos *in natura* ou minimamente processados, ingredientes culinários, alimentos processados e ultraprocessados^16^.

Inicialmente as características dos domicílios participantes da pesquisa (região, área de localização [urbano e rural] e renda familiar *per capita*) foram descritas por meio de frequências absolutas e relativas. A renda familiar *per capita* foi estratificada com base no salário-mínimo vigente em janeiro de 2018 (R$ 954,00): menos de um salário-mínimo, um a dois, dois a três e mais que 4 salários-mínimos.

A frequência de domicílios que adquiriram alimentos em cada um dos locais foi estimada segundo locais de aquisição e nível de processamento dos alimentos adquiridos (*in natura*, ingredientes, processados ou ultraprocessados). Foram considerados, para ambas as estimativas, a aquisição de pelo menos um item no local de aquisição ou um item do grupo alimentar.

Para estimar a frequência de aquisição dos grupos de alimentos segundo locais de aquisição, dividiu-se o número de domicílios que adquiriram pelo menos um alimento de um grupo alimentar em determinado local de aquisição pelo número de domicílios que adquiriram pelo menos um item nesse local.

Os dados foram apresentados em frequência relativa, e a significância das diferenças foi considerada por meio da não sobreposição dos intervalos de confiança de 95% (IC 95%). Todas as estimativas foram estratificadas segundo a localização do domicílio (urbano/rural) e faixas de renda familiar *per capita*, calculadas pelo *software* SAS, e levaram em conta a complexidade do desenho e o peso amostral da pesquisa.

## RESULTADOS

Dos 49.489 domicílios avaliados, 82,3% localizavam-se na área urbana do país; em 51,4% o chefe da família possuía ensino fundamental; e 69,3% apresentaram renda familiar *per capita* de até dois salários-mínimos (Tabela 1).

**Tabela 1:** Caracaterísticas sociodemográficas dos domicílios. Brasil, 2017–2018.

Variáveis	2017–2018
	% (IC95%)
Área de localização	
Urbano	82,3 (85,7–86,8)
Rural	13,7 (13,2–14,2)
Região	
Norte	7,1 (6,9–7,4)
Nordeste	27,7 (27,1–28,3)
Sudeste	42,2 (41,5–43,0)
Sul	15,4 (14,9–15,9)
Centro-Oeste	7,5 (7,1–7,9)
Escolaridade*	
Ensino fundamental	51,4 (50,6–52,3)
Ensino médio	29,6 (29,0–30,3)
Ensino superior	18,9 (18,1–19,8)
Renda (salário mínimo)^†^	
≤1	34,2 (33,5–35,0)
1–2	35,1 (34,4–35,9)
2–4	13,9 (13,4–14,4)
> 4	16,7 (15,8–17,5)

*Escolaridade do chefe da família;

^†^Renda familiar *per capita*. IC: intervalo de confiança.

Os locais de maior contribuição na aquisição de alimentos/bebidas foram supermercado (51,9%), seguido de pequenos mercados (44,5%) e padaria (43,8%). Os centros de distribuição de alimentos/atacadistas tiveram a menor participação (5,5%). Diferenças importantes entre as áreas urbanas e rurais foram encontradas. Para a área urbana, supermercado (55,0%), padaria (46,5%) e pequenos mercados (43,1%) estão entre os locais com maior frequência de aquisição de alimentos, enquanto, para as áreas rurais, verifica-se que pequenos mercados (53%), supermercado (32,3%) e produção em casa (31,0%) apresentaram as maiores frequências (Tabela 2).

**Tabela 2:** Frequência (%) e intervalo de confiança dos locais de aquisição de alimentos/bebidas, segundo área de localização (urbano/rural). Brasil, 2017–2018.

Locais de aquisição*	Brasil (%) (IC95%)	Urbano (%) (IC95%)	Rural (%) (IC95%)
Supermercado	51,9 (51,0–52,8)	55,0 (54,0–56,0)	32,3 (30,5–34,2)
Padaria	43,8 (43,0–44,6)	46,5 (45,6–47,4)	26,7 (25,0–28,3)
Pequenos mercados	44,5 (43,6–45,4)	43,1 (42,1–44,1)	53,0 (50,8–55,1)
Frutaria/feiras/mercado de rua	23,0 (22,3–23,6)	23,2 (22,5–24,0)	21,3 (19,4–23,1)
Açougue/peixaria	16,3 (15,8–16,9)	16,0 (15,4–16,6)	18,5 (17,2–19,4)
Comida de rua/vendedores de rua	9,5 (6,1–9,9)	8,2 (7,8–8,7)	17,5 (15,8–19,3)
Restaurante/lanchonete/bar/cafeteria	7,2 (6,8–7,6)	7,8 (7,3–8,2)	3,8 (3,2–4,3)
Produção em casa	7,0 (6,6–7,4)	3,2 (2,9–3,4)	31,0 (28,7–33,3)
Centros de distribuição de alimentos/atacadistas	5,5 (5,1–5,8)	5,9 (5,5–6,4)	2,4 (1,9–3,0)
Outros	11,2 (10,8–11,7)	10,0 (9,5–10,4)	19,2 (17,8–20,6)

*% de domicílios que adquiriram pelo menos um alimento/bebida desse local. IC: intervalo de confiança.

Com relação à frequência de aquisição de alimentos/bebidas, de acordo com o nível de processamento, 91,6% dos domicílios analisados adquiriram alimentos *in natura*, seguidos de 78,8% da aquisição de processados e de 72,9% de ultraprocessados. Os ingredientes obtiveram apenas 34,8% de contribuição. Observam-se elevados percentuais de frequência de aquisição de alimentos processados e ultraprocessados entre os domicílios da área urbana, e a aquisição de alimentos ultraprocessados está em segundo lugar entre os residentes das áreas rurais do país (Figura 1A). Considerando-se a renda, não houve diferença significativa para a aquisição de alimentos *in natura* e ingredientes nos três primeiros níveis de renda, no entanto a aquisição de processados e ultraprocessados aumenta conforme aumenta a renda (Figura 1B).

**Figura 1:** Frequência (%) da aquisição de alimentos/bebidas, segundo nível de processamento dos alimentos adquiridos. Brasil, 2017–2018.

Os locais de aquisição de alimentos também diferiram conforme o nível de renda, sendo a aquisição em supermercados 29,6 pontos percentuais (pp) maior entre os que recebem mais de quatro salários-mínimos em comparação com os que recebem menos de um. Por outro lado, o percentual de aquisição de alimentos provenientes de padaria, pequenos mercados, açougue/peixaria, comida de rua/vendedores de rua e produção em casa diminuiu com a renda (Tabela 3).

**Tabela 3:** Frequência (%) e intervalo de confiança dos locais de aquisição de alimentos/bebidas, segundo renda familiar *per capita* do domicílio. Brasil, 2017–2018.

Locais de aquisição*	≤1 SM % (IC95%)	1–2 SM % (IC95%)	2–4 SM % (IC95%)	>4 SM % (IC95%)
Supermercado	39,7 (38,5–40,8)	52,3 (51,0–53,6)	60,0 (58,0–62,0)	69,3 (67,5–71,3)
Padaria	47,7 (46,6–48,8)	44,5 (43,3–45,6)	39,4 (37,4–41,5)	37,9 (36,0–39,8)
Pequenos mercados	54,9 (53,7–56,1)	43,0 (41,8–44,2)	38,5 (36,6–40,4)	31,2 (29,2–33,2)
Frutaria/Feiras/Mercado de rua	23,4 (22,4–24,5)	22,5 (21,6–23,5)	22,4 (20,7–24,2)	23,2 (21,6–24,8)
Açougue/Peixaria	19,6 (18,7–20,4)	16,0 (15,2–16,8)	14,4 (13,1–15,7)	12,0 (10,8–13,2)
Comida de rua/vendedores de rua	12,8 (12,0–13,6)	9,2 (8,6–9,8)	7,0 (6,2–7,9)	5,5 (4,8–6,3)
Restaurante/lanchonete/bar/cafeteria	5,3 (4,8–5,8)	7,1 (6,5–7,7)	8,7 (7,5–9,9)	10,2 (9,2–11,3)
Produção em casa	9,2 (8,5–9,8)	7,3 (6,7–7,8)	5,5 (4,8–6,1)	3,1 (2,7–3,6)
Centros de distribuição de alimentos/atacadistas	4,1 (3,7–4,6)	5,5 (4,9–6,1)	6,6 (5,3–7,9)	7,1 (6,2–8,0)
Outros	12,6 (11,9–13,3)	10,7 (10,0–11,3)	9,9 (8,9–10,9)	10,6 (9,5–11,7)

*% de domicílios que adquiriram pelo menos um alimento/bebida desse local. SM; Salário mínimo; IC: intervalo de confiança.

Para as áreas urbanas, grande parte dos alimentos *in natura* foi adquirida no açougue/peixaria (97,5%), frutaria/feiras/mercado de rua (97,1%), produção em casa (95,3%) e supermercados (92,2%). Alimentos processados foram obtidos, majoritariamente, em padaria. No que diz respeito ao grupo dos ultraprocessados, o local que apresentou maior frequência de aquisição foi o supermercado (78,6%) (Tabela 4).

**Tabela 4:** Frequência (%) e intervalo de confiança de aquisição dos grupos de alimentos segundo locais de aquisição na área urbana. Brasil, 2017–2018.

Locais de aquisição*	*In natura* (IC95%)	Ingredientes (IC95%)	Processados (IC95%)	Ultraprocessados (IC95%)
Supermercado	92,2 (91,6–92,7)	37,5 (36,4–38,5)	61,0 (59,9–62,1)	78,6 (77,7–79,5)
Padaria	21,0 (20,0–22,0)	1,0 (0,8–1,2)	95,5 (95,1–96,0)	28,3 (27,3–29,3)
Pequenos mercados	84,4 (83,5–85,2)	26,7 (25,7–27,7)	49,1 (47,9–50,3)	67,1 (66,1–68,1)
Frutaria/feiras/mercado de rua	97,1 (96,6–97,6)	2,5 (2,1–3,0)	10,6 (9,4–11,9)	12,3 (11,2–13,5)
Açougue/peixaria	97,5 (97,0–98,1)	0,4 (0,2–0,6)	3,3 (2,7–3,9)	14,6 (13,2–16,1)
Comida de rua/vendedores de rua	82,1 (80,0–84,3)	0,5 (0,3–0,8)	18,2 (16,0–20,3)	11,6 (9,9–13,4)
Restaurante/lanchonete/bar/cafeteria	43,3 (40,6–46,0)	0,3 (0,0–0,6)	18,6 (16,5–20,7)	60,5 (57,6–63,4)
Produção em casa	95,3 (93,6–96,9)	5,4 (3,5–7,2)	10,2 (8,2–12,3)	8,8 (6,6 –10,9)
Centros de distribuição de alimentos/atacadistas	82,8 (80,3–85,3)	25,1 (22,3–27,8)	40,3 (37,0–43,7)	51,8 (48,5–55,0)
Outros	69,6 (67,4–71,8)	6,0 (5,0–7,0)	23,7 (21,8–25,6)	34,0 (31,9–36,1)

*% de domicílios que adquiriram pelo menos um alimento/bebida desse local. IC: intervalo de confiança.

Na área rural, o principal local de aquisição dos alimentos *in natura* foi produção em casa (98,8%), seguido de açougue/peixaria (97,9%) e frutaria/feiras/mercado de rua (96,5%), mas a aquisição de ingredientes culinários, alimentos processados e ultraprocessados também se deu em grande parte nos supermercados e pequenos mercados (Tabela 5).

**Tabela 5:** Frequência (%) e intervalo de confiança de aquisição dos grupos de alimentos segundo locais de aquisição na área Rural. Brasil, 2017–2018.

Locais de aquisição*	*In natura* (IC95%)	Ingredientes (IC95%)	Processados (IC95%)	Ultraprocessados (IC95%)
Supermercado	90,2 (89,0–91,5)	49,0 (46,6–51,5)	44,6 (42,1–47,2)	74,1 (71,9–76,2)
Padaria	11,7 (9,9–13,5)	1,1 (0,4–1,7)	95,3 (94,3–96,3)	18,8 (16,7–20,9)
Pequenos mercados	85,5 (84,0–87,0)	41,3 (39,3–43,3)	38,4 (36,6–40,5)	64,0 (62,1–65,8)
Frutaria/Feiras/Mercado de rua	96,5 (95,2–97,8)	7,5 (5,5–9,4)	9,6 (7,2–12,0)	14,9 (11,9–17,9)
Açougue/Peixaria	97,9 (97,1–98,6)	0,5 (0,03–1,0)	1,6 (0,7–2,4)	6,7 (5,2–8,3)
Comida de rua/vendedores de rua	69,9 (65,4–74,5)	1,6 (0,8–2,3)	43,2 (37,4–49,1)	10,4 (8,3–12,5)
Restaurante/Lanchonete/Bar/Cafeteria	37,5 (30,6–44,5)	3,7 (1,2–6,1)	29,7 (23,7–35,6)	54,5 (47,8–61,1)
Produção em casa	98,8 (98,3–99,3)	1,7 (1,2–2,3)	6,1 (4,8–7,4)	3,6 (2,7–4,5)
Centros de distribuição de alimentos/atacadistas	85,8 (79,8–91,9)	39,8 (31,7–48,0)	35,9 (26,6–45,2)	43,8 (32,6–55,0)
Outros	86,6 (84,4–88,9)	6,1 (4,7–7,5)	18,0 (15,1–20,8)	16,7 (14,4–18,9)

*% de domicílios que adquiriram pelo menos um alimento/bebida desse local. IC: intervalo de confiança.

## DISCUSSÃO

No presente estudo são descritos os locais de compra, segundo o nível de processamento de alimentos e bebidas e de acordo com a área de localização do domicilio (urbana/rural), com base nos dados da POF realizada em 2017–2018. Os locais que tiveram maior participação na aquisição de alimentos e bebidas foram: supermercados, pequenos mercados e padaria, com diferenças entre as áreas urbanas e rurais. O supermercado manteve-se como o local de maior contribuição para a aquisição nas áreas urbanas, enquanto para as áreas rurais foram os pequenos mercados. A produção em casa está em terceiro lugar em relação aos locais de aquisição das áreas rurais e é dez vezes maior do que a frequência nas áreas urbanas.

Quanto à frequência de aquisição dos grupos de alimentos/bebidas, observa-se que a aquisição de ingredientes culinários, alimentos processados e ultraprocessados se deu em grande parte nos supermercados e pequenos mercados, tanto nas áreas urbanas quanto nas rurais. No entanto, destaca-se para as áreas rurais que o principal local de aquisição dos alimentos *in natura* foi produção em casa.

As diferenças entre as áreas urbanas e rurais do país podem ser reflexo do processo de urbanização das regiões, que influencia o consumo alimentar da população brasileira. Nesse cenário, áreas urbanas demonstram repercussões nas práticas alimentares com maior adesão aos itens processados e ultraprocessados em razão do estilo de vida e da busca pela praticidade dos itens prontos para o consumo. Paralelamente a isso, há uma tendência de integração dos padrões de consumo com o avanço da globalização diante das chances de acesso à informação e disponibilidade de itens industrializados em áreas rurais^17^. A urbanização da área rural, promovida pelo crescente processo de industrialização e ampliada pela produção agroalimentar, tem contribuído para a mudança do estilo de vida e, consequentemente, do padrão alimentar^18^. Análises da tendência temporal das mudanças no consumo de ultraprocessados verificaram aumento da participação desses itens na dieta de indivíduos da área rural em comparação à urbana entre 1974 e 2018 (2,43; IC95% 2,0–2,87 *vs.* 0,6; IC95% 0,23–0,98, respectivamente)^10^.

Diferentes determinantes relacionam-se com os padrões alimentares, que podem ser de natureza física, econômica, política, cultural ou social, não correspondendo somente às questões de escolha individual^4^. Estudos demonstram que a renda está associada a um importante determinante da escolha e dos padrões de aquisição de alimentos, destacando que o acesso e o consumo de alimentos considerados saudáveis são superiores entre os indivíduos de maior nível socioeconômico. Inversamente, indivíduos em maior vulnerabilidade socioeconômica possuem menor acesso a itens saudáveis, como frutas e vegetais, e maior exposição a itens processados e ultraprocessados^19-21^.

Diferenças quanto ao nível de renda também foram observadas em nosso estudo, sendo a frequência de aquisição em supermercado quase 30 pontos percentuais maior nos indivíduos de maior nível socioeconômico em comparação com os de menor nível. Além disso, domicílios com maior renda apresentam maiores frequências de aquisição tanto de alimentos *in natura* como de processados e ultraprocessados. Levy et al.^10^ verificaram que, quanto maior a renda, maior a participação de alimentos ultraprocessados no total de energia consumida. No entanto, ao avaliarem a tendência temporal das mudanças no consumo de ultraprocessados, observaram um aumento mais expressivo na participação da dieta de indivíduos do menor nível socioeconômico.

As desigualdades na disponibilidade e no acesso do ambiente alimentar, no qual os indivíduos estão inseridos, contribuem para o aumento da insegurança alimentar em áreas economicamente desfavorecidas. Isso porque regiões de menor nível socioeconômico são frequentemente caracterizadas como pântanos alimentares, locais onde há maior disponibilidade de estabelecimentos que comercializam itens processados e ultraprocessados. Esse cenário limita o acesso a alimentos considerados marcadores de alimentação saudável e aumenta a exposição a itens cujo consumo regular é amplamente relacionado a desfechos desfavoráveis de saúde e nutrição^22^.

Observamos que o supermercado foi um ambiente de elevada contribuição para a aquisição de ultraprocessados, sugerindo alta disponibilidade desses itens no ambiente do consumidor. Somando-se aos nossos achados, estudos nacionais que avaliaram a relação entre o consumo alimentar e o local de aquisição dos alimentos observaram que o acesso a itens provenientes de supermercados estava associado ao consumo de alimentos de baixa qualidade nutricional, como os ultraprocessados^11,14^. Os pequenos mercados também apresentaram importante contribuição para a aquisição de alimentos e foram associados à disponibilidade de itens ultraprocessados, frequentemente atrativos por serem mais fáceis de armazenar, não necessitando de equipamentos específicos para a comercialização^23^.

A alta aquisição de alimentos ultraprocessados pode estar relacionada tanto com o preço como com a facilidade de consumo e o apelo de promoções. Estudo que buscou relacionar a prevalência de promoções de preços de alimentos e bebidas e sua influência no comportamento de aquisição do consumidor constatou que as promoções de preços eram mais comuns para alimentos e bebidas não saudáveis^24^. Residir em áreas distantes dos locais de compra de alimentos também está associado a um maior consumo de ultraprocessados, enquanto comprar os alimentos em estabelecimentos mais próximos ao domicílio está associado a um padrão alimentar mais saudável^23^. Em contrapartida, morar em áreas onde há feiras e mercados que comercializam frutas, verduras e legumes com boa qualidade torna mais provável a adoção de padrões marcadores de alimentação saudável^3^.

Embora a maioria dos domicílios tenha adquirido alimentos *in natura* (91,6%), também observamos alta aquisição de itens processados e ultraprocessados, 78,8 e 72,9%, respectivamente. Para além da disponibilidade, acessibilidade, conveniência, preço, promoções e qualidade, outros determinantes podem influenciar o comportamento de aquisição de alimentos e a mudança nos padrões de consumo, tais como características socioculturais, conveniência, economia de tempo e esforço e sustentabilidade dos itens em determinados ambientes^25^. Estudos desenvolvidos no Brasil reforçam nossos achados identificando aumento no padrão de consumo de itens marcadores de alimentação saudável e estabilidade no consumo de itens considerados marcadores de alimentação não saudável, como combos de *fast-food*, doces e biscoitos, salgadinhos de pacote e refrigerantes^26^.

A adoção do padrão não saudável pode ser relacionada não apenas às dimensões do ambiente alimentar comunitário, mas à mudança no cotidiano e nas formas de aquisição de comida preparada fora do domicílio. Apesar de restaurante/lanchonete/bar/cafeteria terem apresentado baixa frequência de aquisição, viu-se que a aquisição nesses estabelecimentos foi de alimentos ultraprocessados, o que pode ser um indicativo da aquisição de alimentos prontos para consumo preparados fora do domicílio. A comparação dos dados das POF de 2002–03 a 2017–18 mostra aumento na aquisição de refeições prontas para consumo nos domicílios brasileiros, passando de 0,2 para 0,7%, respectivamente^27^. Esses alimentos podem ser consumidos tanto nos estabelecimentos como dentro do domicílio pela aquisição via *delivery* ou *takeaway* ^8^. Essas práticas de aquisição passaram a integrar o cotidiano dos indivíduos e são consideradas um fator determinante no aumento dos níveis de sobrepeso e obesidade por seu conteúdo nutricional desfavorável^8^.

Mais recentemente, as práticas de aquisição de comida preparada fora do domicílio também apresentaram ascensão para o uso de aplicativos de dispositivos digitais. Estudos acerca da disponibilidade de alimentos no ambiente alimentar digital de aplicativos de entrega de comida parecem mostrar similaridade em relação às características de um pântano alimentar, pela elevada disponibilidade de itens como lanches e combos de *fast-food* e bebidas açucaradas, além do impacto das estratégias de *marketing* e fidelização para influenciar as escolhas do consumidor^28-30^.

As recomendações do *Guia Alimentar para a População Brasileira* orientam que a base da alimentação seja composta de alimentos *in natura* ou minimamente processados, alimentos que proporcionam uma dieta nutricionalmente adequada e saborosa, e reconhecem a relevância dos aspectos culturais e de sustentabilidade que envolvem a alimentação. Incentivam a aquisição de alimentos de feiras livres, produtores locais ou outros lugares de base agroecológica^16^. O fortalecimento da produção de agricultores locais e a valorização dos insumos regionais são estratégias que podem favorecer padrões alimentares mais saudáveis e sustentáveis^31^.

Destacam-se os esforços do governo brasileiro para transpor barreiras de ambientes alimentares do consumidor predominantemente não saudáveis no intuito de ampliar o acesso da população a alimentos mais saudáveis. A modificação da composição da cesta básica brasileira, privilegiando itens *in natura* ou minimamente processados, levando em consideração as necessidades nutricionais das diferentes regiões do país, com foco na agricultura familiar e exclusão de ultraprocessados, é um exemplo. A regulamentação do Programa Cozinha Solidária, iniciativa voltada à distribuição gratuita ou com preços acessíveis de alimentos, é outra estratégia que pode contribuir para a melhoria da segurança alimentar e nutricional e o combate à fome no país, proporcionando acesso a alimentos de melhor qualidade^32^. Por outro lado, mudanças no ambiente requerem políticas públicas intersetoriais^33^. Como exemplos, podemos citar México e Reino Unido, que adotaram políticas de taxação de bebidas açucaradas, e os primeiros resultados no México mostraram que essa mudança provocou redução de 6% na compra dessas bebidas^34,35^.

Dado importante a ser analisado em nossos achados é a categoria de locais de aquisição denominada de “outros”, ou seja, locais que não possuíam como finalidade principal a venda de alimentos. Dessa forma, acredita-se que tais locais comercializem produtos de fácil conservação, como é o caso dos ultraprocessados. Contudo, observou-se que domicílios que adquiriram alimentos nesses locais compraram mais alimentos *in natura*, representando um dado positivo observado no presente estudo. Atualmente, muitos alimentos podem ser adquiridos via internet, contudo este não foi considerado como um local de aquisição específico para esta pesquisa por representar apenas um meio de adquirir o produto, o qual pode ser oriundo de qualquer lugar caracterizado anteriormente, não havendo como distingui-lo, o que representa uma limitação deste estudo. Soma-se a essa limitação a falta de informação detalhada sobre os locais de aquisição, por exemplo se os centros de distribuição são equipamentos públicos de Segurança Alimentar e Nutricional com distribuição/doação de alimentos. No entanto, “centros de distribuição de alimentos” foram reportados e incluídos nesta análise. Além disso, consideramos todas as aquisições monetárias e não monetárias, sendo assim itens adquiridos via doação ou por meio de acesso a benefícios foram incluídos nas análises.

Como ponto forte, este artigo é o mais atual quanto à fonte dos alimentos adquiridos para consumo nos domicílios brasileiros, sendo capaz de traçar um panorama das práticas de aquisição e consumo de alimentos, direcionando a elaboração de estratégias e de intervenções que sejam capazes de proporcionar ambientes alimentares mais saudáveis, melhorar a segurança alimentar e nutricional e prevenir doenças associadas ao estilo de vida^2^.

Em termos de saúde pública, cabe destacar a importância das diferenças observadas entra a área urbana e a rural, indicando a necessidade de estratégias que envolvam multicomponentes no sentido de interromper a inversão das práticas alimentares na área rural e propiciar melhor acesso a alimentos *in natura* e minimamente processados tanto na área rural como na urbana.

Em conclusão, nossos achados sinalizam que a aquisição de alimentos para consumo domiciliar no Brasil vem principalmente de supermercados e pequenos mercados, que apesar de serem fontes de alimentos *in natura* também foram locais de maior percentual na aquisição de alimentos ultraprocessados. Diferenças entre as áreas rurais e urbanas sinalizam a relevância de ações no cenário do ambiente alimentar comunitário, que visem a processos de educação alimentar e nutricional pautados nas recomendações do *Guia Alimentar para a População Brasileira*, nas múltiplas dimensões das escolhas alimentares saudáveis, sustentáveis e que valorizam a cultura alimentar em diferentes cenários comunitários. Assim, pode-se fomentar a adesão ao padrão de consumo de alimentos *in natura* e minimamente processados e desencorajar o consumo de itens ultraprocessados.
